# Trends in equity of inpatient health service utilization for the middle-aged and elderly in China: based on longitudinal data from 2011 to 2018

**DOI:** 10.1186/s12889-021-11203-y

**Published:** 2021-06-16

**Authors:** Xiaojing Fan, Min Su, Yaxin Zhao, Yafei Si, Zhongliang Zhou

**Affiliations:** 1 School of Public Policy and Administration, Xi’an Jiaotong University, Xi’an, China; 2 School of Public Administration, Inner Mongolia University, Hohhot, China; 3 School of Public Health, Xi’an Jiaotong University Health Science Centre, Xi’an, China; 4 School of Risk & Actuarial Studies and CEPAR, University of New South Wales, Kensington, Australia

**Keywords:** Inpatient health service utilization, Equity, Middle-aged and elderly

## Abstract

**Background:**

The aim of this study was to assess the trends in equity of receiving inpatient health service utilization (IHSU) in China over the period 2011–2018.

**Methods:**

Longitudinal data obtained from China Health and Retirement Longitudinal Studies were used to determine trends in receiving IHSU. Concentration curves, concentration indices, and horizontal inequity indices were applied to evaluate the trends in equity of IHSU.

**Results:**

This study showed that the annual rate of IHSU gradually increased from 7.99% in 2011 to 18.63% in 2018. Logistic regression shows that the rates of annual IHSU in 2018 were nearly 3 times (OR = 2.86, 95%CL: 2.57, 3.19) higher for rural respondents and 2.5 times (OR = 2.49, 95%CL: 1.99, 3.11) higher for urban respondents than the rates in 2011 after adjusting for other variables. Concentration curves both in urban and rural respondents lay above the line of equality from 2011 to 2018. The concentration index remained negative and increased significantly from − 0.0147 (95% CL: − 0.0506, 0.0211) to − 0.0676 (95% CL: − 0.0894, − 0.458), the adjusted concentration index kept the same tendency. The horizontal inequity index was positive in 2011 but became negative from 2013 to 2018, evidencing a pro-low-economic inequity trend.

**Conclusions:**

We find that the inequity of IHSU for the middle-aged and elderly increased over the past 10 years, becoming more focused on the lower-economic population. Economic status, lifestyle factors were the main contributors to the pro-low-economic inequity. Health policies to allocate resources and services are needed to satisfy the needs of the middle-aged and elderly.

## Background

Equity in health service utilization, as a significant outcome of health systems, has always attracted extensive research interest [1–8]. Health services should be equitably distributed, whereby people who have greater need should use health services proportionally more than those with less need [9]. However, studies have shown that there were differences in health service utilization, even with the same need, whereby individuals with higher economic status, greater education, and health insurance utilize more health services [2, 3, 10].

As a rapidly industrializing middle-income country, the widening income-related inequity in access to health services among residents has been a growing concern of the Chinese government. In response to the growing inequalities in healthcare utilization, the Chinese government has made substantial effort since 2002 to reduce the burden of inpatient costs and make healthcare more affordable for patients. In 2009, the Chinese government launched a new healthcare reform policy with the goal of effectively reducing the burden of medical expenses and providing affordable universal healthcare for all citizens [11–15]. In 2011, the Chinese government set a target to reimburse approximately 70% of inpatient expenses. In 2013, the National Health Commission increased the rate of reimbursement expense for inpatient health service to around 75% for rural residents. These health policies that favor people at lower income levels have made significant progress in the provision of health services in recent decades by treating affordability as a major barrier to equal access to health services. For the middle-aged and elderly, we also assume that their inpatient health service utilization (IHSU) is gradually increasing recently. However, although the inequality in the utilization of health services among middle-aged and elderly still exists and has formed a relatively serious social problem, there are relatively few studies on the latest trends in their IHSU. Middle-aged and elderly population bear a higher burden of chronic diseases, depression, disability and other diseases than young people as they grow older, which leads to a greater demand for medical services, especially inpatient care, it is essential to monitor the changes in IHSU rates.

Previous studies in China have provided a rich foundation for the study of IHSU. Zhu et al. analyzed both regional and individual socioeconomic factors associated with the inequality of outpatient and inpatient service utilization in China, using data from 1991 to 2011, and they provided evidence to improve governmental health policies [2]. Some scholars have suggested that inequality should be reduced by reducing the income gap between the rich and the poor through social risk protection after a study on the equity of IHSU in rural areas of China [3]. Some scholars have also studied the equity of IHSU among the elderly and found that there is an inequality that tends to be higher-economic groups, while the New Rural Cooperative Medical Scheme is less effective in reducing this inequality [16]. In addition, the National Health Service survey of China has showed a gradually enhancing trend in IHSU rate (from 6.8% in 2008 to 9.0% in 2013) for the all-age population [17, 18]. Our study aims to update the information on the trends in annual rates and equity of IHSU among an older segment of the population-the middle-aged and elderly, to quantify personal socioeconomic factors associated with inequalities in IHSU in China, and to provide scientific evidence and recommendations for improving governmental health policies concerning healthcare inequity.

## Methods

### Data

**Fig. 1:**
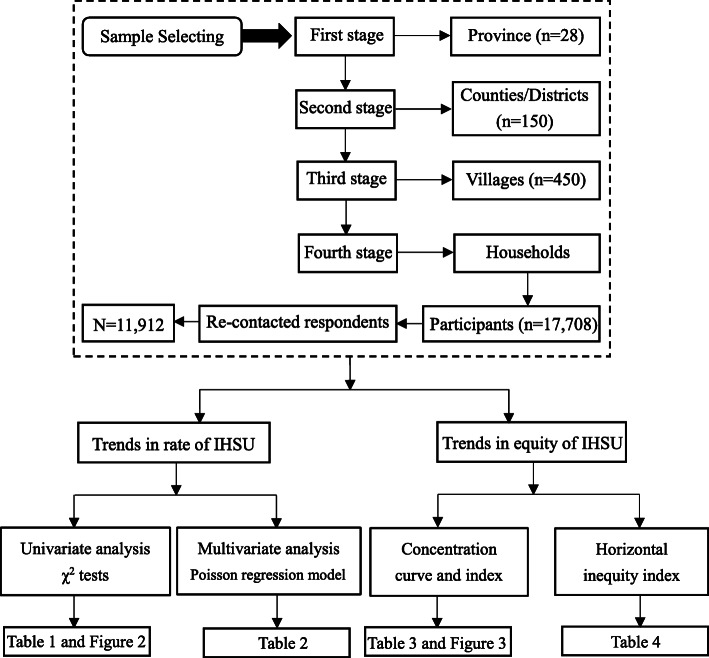
Flowchart on the sample selecting process at each step and analysis framework

Data were drawn from the 2011, 2013, 2015, and 2018 China Health and Retirement Longitudinal Studies (CHARLS), which used a structured questionnaire with a longitudinal large-scale nationally representative sample of people aged 45 years or older (the data and questionnaire are available at http://charls.pku.edu.cn/) [19]. Employing four-stage stratified sampling, CHARLS was implemented in 2011 to cover 28 provinces in China and investigated a sample of 17,708 participants in about 10,257 households in 150 counties/districts [20]. The baseline data collection was performed in 2011 with an overall response rate of 80.5%, and then three follow-up interviews were conducted in 2013, 2015 and 2018. In the study, 11,912 re-contacted respondents were enrolled. The detailed process is shown in Fig. 1.

### Measures

CHARLS baseline data included detailed information of respondents and their living spouses. The main questionnaire included information on basic demographics, family, health status, healthcare and health insurance, and household and individual economy. The “healthcare and health insurance” section in the questionnaire provided the most important information for this study. The outcome variable, IHSU, was based on respondents’ reports from the 2011–2018 waves on the following item: “Have you received inpatient care in the past year?” This was a binary variable, whereby 0 denoted “no” and 1 denoted “yes”. The key independent variables included respondents’ gender, age, education, insurance, income, living status, sleeping hours, smoking status, alcohol consumption, and health status taken from the 2011 wave of CHARLS. Living place was divided into rural and urban groups. Respondents’ education was coded into two levels: (1) lower than elementary school, and (2) high school and above. Household income per capita was a continuous variable, calculated as the sum of personal assets, household assets, personal income and income from other household members, household income from agriculture, household income from self-employment, and household income from transfers divided by the total number of family members in the household. It was further divided into three groups. In order to make inference to the whole population in China, we considered individual weight with household and individual non-response adjustment at baseline, the adjusted data were included in the CHARLS data.

Considering the fact that there are huge socioeconomic gaps between rural and urban areas in China, rural residents are more likely to encounter barriers (inadequate healthcare insurance coverage, long distances to healthcare facilities, lack of transportation, and an undersupply of particular specialists) to receiving health services than people in urban areas [2, 21–24]. Accordingly, the rural and urban samples were analyzed separately.

### Health inequity

Concentration curves, concentration indices, and their decomposition were applied to analyze the equity of IHSU. Concentration curves and indices were used to measure the extent of economic status-related inequality in the distribution of IHSU across the population [25, 26]. A positive concentration index denotes that people with high economic status use more IHSU than their low-economic counterparts, whereas a negative index denotes the opposite. The CI formula was as follows:

$$ C=\frac{2}{\upmu} COV\left(y,\gamma \right), $$

where C is defined in terms of the covariance between the outcome variable (y) and the fractional ranks of household income (γ); μ is the mean of y. The outcome variable IHSU in this study is a binary variable, Wagstaff proposed a method for correcting CI [26], the formula as follows:

$$ {\mathrm{C}}_{\mathrm{adjusted}}=\frac{1}{1-\upmu}C $$

Inequality can be further explained by decomposing the concentration index into its determining components; then, the horizontal inequity index (HI) can be computed by subtracting the contribution of variables (such as sex, age, health status) from the concentration index of IHSU [9]. These determinants were selected according to previous research, constrained by the variables collected in the investigation [27, 28]. A probit regression model was used to indirectly standardize the IHSU since the outcome variable was binary.

### Analytical strategy

Categorical variables were presented as absolute numbers as a proportion of the total number of participants. Chi-square tests were used to separately test whether the rates of IHSU were statistically significant for the rural and urban samples. Logistic regression was employed to analyze the adjusted odds ratios (ORs) for IHSU after controlling for a number of confounding factors at baseline, such as the year (2011, 2013, 2015, and 2018), sex, age, education, insurance, household income per capita, living status, sleeping hours, smoking and drinking status, and having a disability and chronic disease.

All statistical analyses were performed using STATA statistical software version 14.0 (StataCorp LP, College station 77,845, USA). A two-tailed *p*-value < 0.05 was considered statistically significant.

## Results

### Descriptive statistics

According to the descriptive statistics in Table 1, 46.28% of the middle-aged and elderly were male and 53.72% were female. For rural respondents, 64.25% of them were aged between 45 and 60 years, while this age range constituted 57.60% of the urban cohort. More rural respondents were educated below elementary-school level (74.50%), while more urban respondents were educated above middle-school level (62.07%). Furthermore, 92.80% of rural respondents and 84.04% of urban respondents had insurance. The majority of respondents in all groups were living with others, sleeping more than 7 h, and not smoking and drinking. Only 17.79% of the rural middle-aged and elderly had a disability, whereas 33.14% of them had a chronic disease; on the other hand, 12.79% of the urban middle-aged and elderly had a disability, whereas 33.14% of them had a chronic disease.

**Table 1:** Basic characteristics of respondents in 2011 (*n* = 11,912)

Variables	Group	Rural (***n*** = 9808)	Urban (***n*** = 2092)	***P***
**Demographics**				
Sex	Male	4423(45.13)	1081(51.67)	< 0.001
	Female	5377(54.86)	1011(48.33)	
Age (years)	45–50	2477(25.25)	443(21.18)	< 0.001
	51–60	3825(39.00)	760(36.32)	
	61–70	2531(25.81)	615(29.40)	
	≥71	975(9.95)	274(13.10)	
Education	≤Elementary school	7302(74.50)	792(37.93)	< 0.001
	≥Middle school	2499(25.50)	1296(62.07)	
Insurance	No	702(7.20)	330(15.96)	< 0.001
	Yes	9045(92.80)	1738(84.04)	
Household income per capita (CNY)	Low	2595(27.11)	297(14.86)	< 0.001
	Medium	5072(52.98)	714(35.72)	
	High	1906(19.91)	988(49.42)	
**Life style**				
Living status	Live with others	8122(82.81)	1819(86.95)	< 0.001
	Live alone	1686(17.19)	273(13.05)	
Sleeping hours	7–8 h	3762(38.36)	883(42.21)	< 0.001
	≤6 h	4576(46.66)	980(46.85)	
	> 8 h	1470(14.98)	229(10.95)	
Smoking status	Yes	3768(38.53)	787(37.84)	0.554
	No	6011(61.47)	1293(62.16)	
Alcohol consumption	Yes	3215(32.89)	693(33.32)	0.709
	No	6559(67.11)	1387(66.68)	
**Health status**				
Disability	No	8035(82.21)	1815(87.22)	< 0.001
	Yes	1739(17.79)	266(12.78)	
Chronic disease	No	3205(33.14)	615(29.70)	0.002
	Yes	6467(66.86)	1456(70.30)	

### Tendency of rate of IHSU

**Fig. 2:**
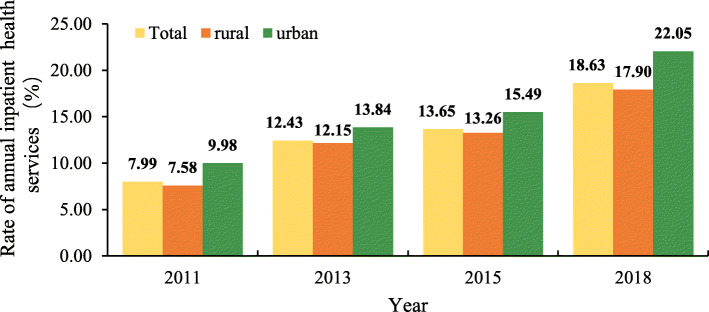
The rate of annual inpatient health service utilization from 2011 to 2018

Figure 2 presents the rate of annual IHSU from 2011 to 2018. In total, the proportion of urban respondents receiving IHSU was higher than that of rural respondents. With regard to urban respondents, 9.98% of respondents received IHSU in 2011; this value slightly increased to 13.84% in 2013 and 15.49% in 2015, and more than doubled in 2018 (22.05%). Compared to 2011, the proportion of rural respondents that received IHSU increased to 17.90%, more than three times the value in 2011 (7.58%).

A specific, logistic regression showed that the rates of annual IHSU in 2018 were nearly 3.0 times (Odds ratio (OR) = 2.86, 95% confidence limits (CL):2.57, 3.19) higher for rural respondents and 2.5 times (OR = 2.49, 95%CL:1.99, 3.11) higher for urban respondents than those in 2011 (after adjusting for respondents’ sex, age, education, insurance, household income per capita, living status, sleeping hours, smoking status, alcohol consumption, and having a disability or chronic disease, Table 2). Urban and rural respondents with a higher age, no drinking, disability, and chronic disease had higher rates of annual IHSU after adjusting for other characteristics (*p* < 0.05; Table 2).

**Table 2:** Determinants of inpatient health services utilization by logistic regression (*n* = 11,912)

Variables	Group	Rural(*n* = 9808)			Urban(*n* = 2092)		
		OR	95%CL		OR	95%CL	
			Lower	Upper		Lower	Upper
**Demographics**							
Time (year)	2011	1.00			1.00		
	2013	1.84	1.63	2.08	1.55	1.22	1.96
	2015	1.90	1.70	2.12	1.85	1.47	2.34
	2018	2.86	2.57	3.19	2.49	1.99	3.11
Sex	Male	1.00			1.00		
	Female	0.94	0.85	1.04	0.93	0.75	1.16
Age (years)	45–50	1.00			1.00		
	51–60	1.10	0.98	1.23	1.68	1.30	2.15
	61–70	1.43	1.27	1.60	2.14	1.66	2.77
	≥71	1.70	1.48	1.96	3.10	2.29	4.19
Education	≤Elementary school	1.00			1.00		
	≥Middle school	1.00	0.89	1.11	1.02	0.87	1.21
Insurance	No	1.00			1.00		
	Yes	1.13	0.99	1.30	1.20	0.96	1.51
Household income per capita (CNY)	Low	1.00			1.00		
	Medium	0.92	0.83	1.01	1.18	0.92	1.51
	High	0.75	0.66	0.85	1.08	0.85	1.37
**Life style**							
Living status	Live with others	1.00			1.00		
	Live alone	1.03	0.93	1.14	0.76	0.59	0.98
Sleeping hours	7–8 h	1.00			1.00		
	≤6 h	1.14	1.06	1.24	1.17	0.99	1.37
	> 8 h	0.96	0.86	1.08	1.07	0.82	1.40
Smoking status	Yes	1.00			1.00		
	No	1.11	1.01	1.21	0.95	0.77	1.18
Alcohol consumption	Yes	1.00			1.00		
	No	1.18	1.07	1.29	1.26	1.04	1.52
**Health status**							
Disability	No	1.00			1.00		
	Yes	1.05	0.96	1.15	1.16	0.94	1.42
Chronic disease	No	1.00			1.00		
	Yes	1.93	1.77	2.11	1.80	1.48	2.19

### Tendency of equity in IHSU

**Fig. 3:**
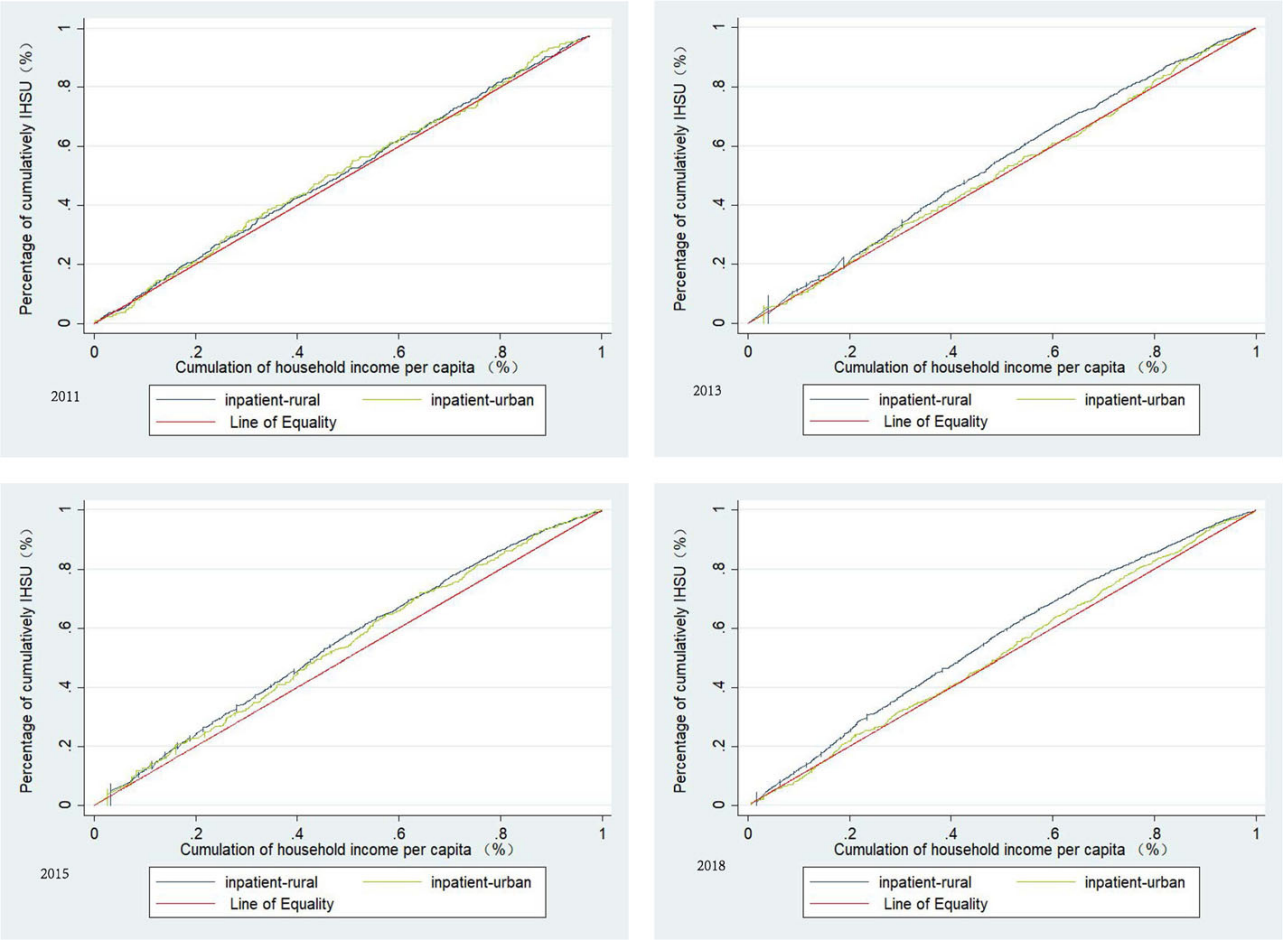
Concentration curves on inpatient health service utilization from 2011 to 2018

Figure 3 shows that, from 2011 to 2018, concentration curves for both urban and rural respondents lay above the line of equality, indicating that the utilization of IHSU was more concentrated among the low-economic respondents. In total, the CI for receiving IHSU remained negative and increased significantly from − 0.2000 to − 0.4459. For rural respondents, this increasing trend was shown from 2011 to 2018; specifically, the adjusted CI was − 0.4240 in 2011, and − 0.7512 in 2018. For urban respondents, the adjusted CI was decreased from − 0.3016 in 2011 to − 0.1536 in 2018 (Table 3).

**Table 3:** Inequality of annual inpatient health services utilization for respondents from 2011 to 2018

Time	Total (*n* = 11,912)				Rural (*n* = 9808)				Urban (*n* = 2092)			
	CI	95% CL		CI _**a**_	CI	95% CL		CI _**a**_	CI	95% CL		CI _**a**_
		Lower	Upper			Lower	Upper			Lower	Upper	
2011	−0.0147	− 0.0506	0.0211	− 0.2000	− 0.0297	− 0.0705	0.0107	− 0.4240	− 0.0271	− 0.1043	0.0491	− 0.3016
2013	− 0.0449	− 0.0726	− 0.0173	− 0.4125	− 0.0665	− 0.0975	− 0.0355	− 0.6230	− 0.0124	− 0.0743	0.0494	−0.1040
2015	−0.0777	− 0.1040	− 0.0514	− 0.6592	− 0.0927	− 0.1222	− 0.0631	− 0.8060	− 0.0716	− 0.1297	− 0.0134	− 0.5470
2018	− 0.0676	− 0.0894	− 0.0458	− 0.4459	− 0.1104	− 0.1352	− 0.0855	− 0.7512	− 0.0264	− 0.0730	0.0202	− 0.1536

Abbreviations: *CI* Concentration Index; *CL* Confidence Limits; *CI*_*a*_ means the adjusted CI

Table 4 presents the detailed information on elasticity, contributions of each determinant to CI and pure percentage contributions of each determinants. A positive elasticity, such as health status indicated having chronic disease was significantly associated with the occurring of IHSU, whilst a negative elasticity, such as living alone, decreased the occurring of IHSU. A positive (negative) contribution denotes that the variable raised (reduced) the pro-rich inequality. We found that age, high economic status, and having a chronic disease had the largest (21.06%), second largest (17.39%), and third largest (9.77%) contributions, respectively, to the inequality of IHSU in 2018. In addition, we found lifestyle had positive elasticity and contributed 17.39% to the inequality of IHSU. Among lifestyle variables, non-smoking and non-alcohol consumption contributed 3.31 and 4.99% respectively to inequality in the 2018 IHSU. In total, the HI of IHSU was positive in 2011 (0.0138) but became negative from 2013 to 2018 (2013:-0.0327; 2015:-0.0401; 2018:-0.0514), the adjusted HI kept the same tendency from 2011 to 2018, evidencing a pro-poor inequity.

**Table 4:** Decomposition analysis of concentration index on respondents’ annual rate of inpatient health services utilization from 2011 to 2018 (*n* = 11,912)

Variables	2011			2013			2015			2018		
	Elasticity	Contribution to CI	%	Elasticity	Contribution to CI	%	Elasticity	Contribution to CI	%	Elasticity	Contribution to CI	%
**Demographics**												
Female	−0.1423	0.0003	− 1.97	− 0.1684	0.0044	−9.81	− 0.0143	0.0004	− 0.56	− 0.1919	0.0066	−9.74
Age (years)												
51–60	0.0647	0.0012	−8.13	0.0688	0.0003	−0.77	0.0948	0.0032	−4.14	0.0680	0.0023	−3.33
61–70	0.1294	−0.0084	57.12	0.0880	−0.0019	4.27	0.1472	−0.0120	15.42	0.1027	−0.0064	9.47
≥ 71	0.0622	−0.0058	39.19	0.0509	−0.0058	13.01	0.0847	−0.0200	25.78	0.0809	−0.0101	14.92
Educated above middle school	0.1172	0.0048	−32.63	−0.0167	−0.0008	1.86	0.0253	0.0013	−1.72	0.0117	0.0008	−1.11
Educated above middle school Insurance (Yes)	0.1148	−0.0006	4.02	0.0888	−0.0003	0.58	0.1055	0.0000	−0.01	−0.0177	0.0001	−0.19
Educated above middle school Household income per capita (CNY)												
Medium	0.0075	0.0000	0.00	−0.0265	0.0006	−1.42	0.0050	−0.0001	0.17	−0.0381	0.0016	−2.35
High	−0.0001	−0.0001	0.76	−0.0307	− 0.0071	15.77	− 0.0003	−0.0001	0.10	−0.0478	− 0.0133	19.74
**Life style**												
Living alone	−0.1203	0.0006	−3.75	−0.0246	0.0001	−0.23	0.0438	−0.0008	1.00	−0.0134	0.0003	−0.40
Sleeping hours												
≤ 6 h	0.0524	− 0.0019	12.95	0.0788	−0.0014	3.09	0.0238	−0.0011	1.46	0.0320	−0.0013	1.85
> 8 h	−0.0031	0.0001	−0.54	− 0.0107	0.0010	−2.15	0.0052	0.0000	0.02	0.0123	−0.0001	0.18
No smoking	0.0880	0.0005	−3.36	0.1150	−0.0011	2.48	0.0846	−0.0012	1.58	0.1456	−0.0022	3.31
No alcohol consumption	0.4503	−0.0019	12.94	0.3800	−0.0053	11.78	0.1351	−0.0024	3.08	0.1831	−0.0034	4.99
**Health status**												
Having disability	0.0316	−0.0049	33.44	0.0162	−0.0024	5.37	0.0041	−0.0007	0.84	0.0110	−0.0020	2.95
Having chronic disease	0.4623	−0.0109	73.87	0.3660	−0.0068	15.13	0.2999	−0.0085	10.88	0.2995	−0.0066	9.77
**HI**	0.0138			−0.0327			−0.0401			− 0.0514		
**HI**_**a**_	0.1877			−0.3004			−0.3402			− 0.3391		

Abbreviations: *CI* Concentration Index; *%* Pure percentage contributions of determinants to the socioeconomic inequality in inpatient health services utilization; *HI* Horizontal inequity index. Needs variables mean contribution of factors to *CI*, including sex, age, disability and chronic disease

## Discussion

This study updates the knowledge on the trends in equity of IHSU for the middle-aged and elderly in China in two ways. First, we used large nationally representative longitudinal survey data in CHARLS to evaluate the level of health utilization from 2011 to 2018; thus, the findings are more generalizable to a wider population in China and might help suggest a more convincing trend of IHSU. Second, this study conducted detailed decomposition analysis of the concentration index for respondents’ IHSU from 2011 to 2018, facilitating the identification of an effective way to reduce the inequity.

According to the National Health Service survey of China in 2008 and 2013, the IHSU rate of the whole population increased from 6.8 to 9.0%, showing a gradually enhancing trend [17, 18]. In the present study, we did observe that the level of IHSU among the middle-aged and elderly in China increased from 2011 to 2018. This phenomenon is consistent with our hypothesis and is also consistent with the other studies among the elderly in China [16, 29]. The medical security system is known to have a positive impact on IHSU; accordingly, the increasing rate of IHSU may be related to the massive increase in demand for medical care following the introduction of medical security systems [30, 31].

The horizontal inequity index changed from 0.0138 in 2011 to − 0.0514 in 2018, suggesting a pro-poor inequity in IHSU, whereby respondents with a low economic status had more IHSU than their high-economic counterparts over the past 10 years. Many studies have confirmed that some demographic or socioeconomic determinants will affect people’s use of inpatient services [32–34]. In our study, age, chronic diseases, economic status and lifestyle were found to be associated with the IHSU, conforming to previous study analyzing health inequity [29]. First of all, age and chronic diseases had positive elasticity and pure percentage contribution to CI, which indicated older and having chronic diseases were significantly associated with the occurring of IHSU. This is not difficult to understand because, as people age, their physical functions decline and they require more health services, especially when it comes to the more vulnerable older segment of the population. According to the fifth National Health Service Survey in China, the prevalence of chronic diseases is increasing year by year, with the majority of the population aged 55 and above [35]. Therefore, the elderly with chronic diseases needs more inpatient services. In addition, our basic health insurance reimburses a greater percent-age of inpatient health services, which may lead older people to use inpatient health services for the treatment of chronic diseases.

Secondly, in addition to the contribution of these need variables, we found the economic status had positive elasticity and pure percentage contribution to CI, it contributed 17.39% to the inequality of IHSU in 2018, which is consistent with previous studies [2, 3, 36]. People with better economic status were more likely to use inpatient health services. One possible explanation is that middle-aged and elderly in better economic situations may have higher expectations for their health and spend more money to obtain higher quality inpatient care. Therefore, health policymakers should consider the key factors that affect equity when allocating health care resources, services, and developing related interventions to meet the diverse health needs of different older population.

Thirdly, among lifestyle variables, we found non-smoking middle-aged and elderly people of lower economic status have more contribution to inequitable outcome for the poor, they use more IHSU. One explanation might be the health problems that led the middle-aged and elderly people to quit smoking, to long-term use of health services thereafter, because previous studies found the former smokers have higher probability of treatments and hospitalization, rehabilitation and the use of medications [37]. Therefore, policy makers need to take into account the health needs of middle-aged and elderly people who have quit smoking in the lower economic levels when formulating future health service related policies. In terms of alcohol consumption, we found non-alcoholic middle-aged and elderly people of lower economic status use more IHSU, which is inconsistent with other researches [38, 39]. Some authors found participants who reduced alcohol consumption to low or completely stop its consumption reported fewer health services utilization [38]. Meanwhile, other authors found no evidence of an association between alcohol consumption and the use of long-term care by older people [39]. More research is needed to explore and verify this association.

This study had several limitations. First of all, all the data were collected via a self-reporting approach and, as such, there may be recall bias. Additionally, the availability of measured determinants of IHSU was limited by the pre-specified questions in the survey, and there could be some potential unobserved confounding factors for which we did not control. Furthermore, although this analysis covered IHSU in 2011, 2013, 2015, and 2018, it was not continuous; hence, the data may not be comprehensive enough to identify the declining level of IHSU and the changes in equity of IHSU. As continuous waves are to be added in the future, it will be important to reexamine these trends.

## Conclusions

The results from this study showed that the urban and rural middle-aged and elderly utilized more inpatient health services over the past 10 years, with greater focus on the lower-economic population. In addition to age > 60 years and having a chronic disease, economic status and lifestyle factors were the main contributors to the pro-low-economic inequity. Health policies to allocate resources and services are needed to satisfy the needs for the middle-aged and elderly, and additional strategies are needed to further reduce the socioeconomic differences in health service utilization.

## Data Availability

The datasets and questionnaire are available at http://charls.pku.edu.cn/.

## Abbreviations

CHARLS: China Health and Retirement Longitudinal Studies
CL: Confidence Limits
CI: Concentration Index
HI: Horizontal inequity index
IHSU: Inpatient Health Service Utilization
OR: Odds Ratio
